# Comparative Analysis of Dried Water Bamboo Shoots Using Different Drying Methods: Physicochemical Properties and Flavor

**DOI:** 10.3390/foods14244357

**Published:** 2025-12-18

**Authors:** Xiaoyang Tong, Kai Zhu, Songheng Wu, Xiaomei Liu, Chenxia Liu, Jun Wang, Hongru Liu, Bingjie Chen, Xiao Wang, Yingdong Jiang, Yongjin Qiao, Yi Zhang

**Affiliations:** 1 Crop Breeding & Cultivation Research Institute, Shanghai Academy of Agricultural Sciences, Shanghai 201403, China; qq3323702770@163.com (X.T.); zhukai2000hnau@163.com (K.Z.); wsh_magnus@163.com (S.W.); l2961852572@163.com (X.L.); liuchenxia@saas.sh.cn (C.L.); 15722469132@163.com (J.W.); hear2008dream@163.com (H.L.); chenbingjie0204@126.com (B.C.); wangxiao.0127@163.com (X.W.); 2 Shanghai Shundi Food Co., Ltd., Shanghai 200333, China; jayson@xiangshangxiang.com

**Keywords:** water bamboo shoots, drying method, quality, volatile organic compound

## Abstract

Drying is a widely utilized method for extending the shelf life of food products. This study applied four drying techniques—natural air drying (NAD), hot air drying (HAD), microwave drying (MD), and vacuum freeze drying (VFD)—to dehydrate water bamboo shoots (WBS), and investigated their effects on the quality and volatile organic compound (VOC) profiles of the dried WBS. The results showed that MD achieved the fastest drying rate, whereas NAD exhibited the slowest. Both MD and VFD samples possessed porous structures. However, the VFD-treated samples retained the highest levels of Vitamin C (VC) and total phenols, and showed the least color deviation compared to the fresh samples, which was closely associated with its low-temperature and vacuum processing conditions. A total of 52 VOCs were identified in this study. Among them, 3-methyl-butanal and 5-methyl-2-furfural were the most abundant VOCs in the treated samples. 3-methybutanal, pentanal, butanal, and 5-methyl-2-furfural were identified as the characteristic VOC markers for the HAD, NAD, VFD, and MD groups, respectively. In conclusion, this study determined that VFD effectively maintained the drying quality of WBS, with butanal identified as its characteristic VOC organic compound. These findings provide valuable insights for the practical production of high-quality dried WBS.

## 1. Introduction

Water bamboo shoots (WBSs), also known as “jiaobai”, are important aquatic vegetables with notable health benefits. WBSs are rich in functional components such as polysaccharides, resistant starch, dietary fiber, vitamins, minerals, flavonoids, and phenolic compounds, which contribute to its antioxidant, anti-atherosclerotic properties and potential role in alleviating alcohol-related conditions [1]. WBSs can be prepared in various ways such as cold dishes or stir-frying, usually using fresh WBSs as the main ingredient. However, the production of WBSs exhibits strong seasonality, which poses a significant constraint on the development of the WBS industry. Although preservation technologies for vegetables have advanced significantly, the high moisture content and tendency toward lignification in WBSs continue to impede its long-term storage [2]. Therefore, drying is an indispensable method for water bamboo shoot preservation. However, commercially available dried water bamboo shoots often exhibit undesirable browning and substantial degradation of functional nutrients. Consequently, it is imperative to explore drying methods capable of enhancing the final product quality.

The development history of drying technology is extensive. From early natural drying methods to modern, highly efficient techniques, the field has undergone a long and diverse evolutionary process. Therefore, dried WBSs can be achieved through various methods, including natural air drying, hot air drying, microwave drying, and vacuum freeze drying. These drying technologies differ in their underlying technical principles, which directly influence their respective advantages. Natural air drying and hot air drying rely on temperature and airflow to promote water evaporation from the surface of the material, thereby achieving the desired drying effect [3]. Microwave drying utilizes high-frequency electromagnetic waves to make the water molecules vibrate at high frequencies within the material, thereby generating internal heat and promoting efficient evaporation [4]. Vacuum freeze drying establishes a low-temperature and vacuum environment for the material and converts the water directly from solid to gas through sublimation, effectively achieving dehydration [5]. Although these four drying techniques are not new technologies, they remain widely applied in industrial settings and continue to serve as the mainstream methods for vegetable drying. Green peppers are commonly dried by hot air to prolong their shelf life; however, this method can induce color changes and non-enzymatic browning, while also reducing the content of important compounds such as chlorophyll, ascorbic acid, and polyphenols [6]. Microwaves are extensively applied in the dehydration of vegetables such as carrots [7], potatoes [8], and cabbage [9]. However, due to the non-uniform distribution of the microwave field within the drying chamber, and the differences in the shape and thickness of vegetables lead to problems such as uneven drying and challenges in controlling drying time [10]. Vacuum freeze drying is characterized by its ability to preserve the structural integrity of materials and minimize nutrient loss. It has been widely applied in the processing of fruit and vegetable products, such as Chinese cabbage [5], wolfberry [11] and mulberry [12]. However, the high time and energy consumption of vacuum freeze drying limit the further promotion of its industry. Currently, research on dried WBSs remains limited, which poses a challenge to meeting the growing market demand for this product. Therefore, this study aims to apply various drying techniques to produce dried WBS, investigate the resulting quality differences, and identify the most appropriate drying method for WBS processing.

The drying quality of vegetables is an important screening criterion for appropriate drying technology, among which the retention rate of functional nutrients is a key indicator. WBSs are known to provide abundant vitamin C and phenolic compounds, which exert antioxidant, anti-inflammatory, and cardiovascular protective effects in the human body [13]. However, during the drying process, functional nutrients will inevitably be degraded, thereby diminishing both the nutritional and economic value of the product. Therefore, it is essential to identify drying techniques that are well-suited for WBS processing. In addition to conventional drying quality evaluation indicators such as rehydration ratio, microstructure, and magnetic relaxation time, the aroma of dried vegetables also significantly influences consumers’ purchasing decisions. Gas chromatography–ion mobility spectrometry (GC-IMS) is a flavor visualization detection technology with the advantages of no pretreatment of samples and rapid detection speed [14]. Currently, GC-IMS is widely applied in food quality research, including the detection of food adulteration, classification, and identification of characteristic volatile organic compounds (VOCs) [15]. This study used GC-IMS technology to investigate the VOC profiles of dried WBSs and determine the effects of different drying technologies on the characteristic aroma of WBS.

Therefore, the purpose of this study was to dehydrate WBSs using four drying methods, natural drying, hot air drying, microwave drying, and vacuum freeze drying, and analyze the changes in functional nutrients, VOCs, and other physical quality parameters. The findings aim to identify the most suitable drying technology, thereby providing a theoretical basis for optimizing the drying process and enhancing the overall quality of WBS.

## 2. Materials and Methods

### 2.1. Sample Collection

The large white water bamboo shoots (purchased from Shanghai Jiaxin Jiaobai Professional Cooperative, Shanghai, China) harvested in August were used as experimental samples. Fresh WBSs were peeled and cleaned, cut into 10 mm × 10 mm × 50 mm cuboids, and randomly divided into 5 groups. The CR group was set as a fresh sample without any treatment. In the NAD group, an electric hot-air drying oven (DHG9240, Shaoxing Supo Instrument Co., Ltd., Shaoxing, China) was used to simulate the natural air drying of WBSs, with the air inlet velocity set to 1 m/s and the air inlet temperature controlled at 35 ± 1 °C. The HAD group used an electric hot-air drying oven (DHG9240, Shaoxing Supo Instrument Co., Ltd.) to dry the WBSs with hot air, with the air inlet speed set at 1 m/s and the air inlet temperature controlled at 75 ± 1 °C. Based on the microwave oven’s (M22J, Midea Group Co., Ltd., Foshan, China) rated power of 700 W, the loading capacity per tray of WBSs was set to 280 g with 1 cm spacing to maintain a microwave drying intensity of 2.5 W/g. In the VFD group, WBSs were freeze-dried using a vacuum freeze dryer (FreeZone^®^ 8 L, Labconco Freeze Dryer Instruments, Kansas City, MO, US) with a vacuum degree of 0.005 mbar and a cold trap temperature of −50 °C. WBSs needed to be frozen at −18 °C before vacuum freeze drying. The treatment was terminated when the moisture content of WBSs in each treatment group dropped to 5% (wet basis). In addition, the WBS samples were not bleached throughout the entire experimental process.

### 2.2. Determination of Moisture Content

The moisture content was analyzed according to previously established methods [16]. A total of 10 g of sample was weighed and placed in an electric hot-air drying oven (DHG9240, Shaoxing Supo Instrument Co., Ltd.) at 105 ± 2 °C for 4 h. Formulas (1) and (2) were used to calculate the wet basis moisture content and dry basis moisture content of WBSs.

(1) $$X\left(\%\right)=\frac{m_{1}-m_{2}}{m_{1}}\;\times \;100\%$$

(2) $$X_{i}\left(\%\right)=\frac{m_{i}-m_{0}}{m_{0}}\;\times 100\%$$

Note: X is the wet basis moisture content, %; X_i_ was the dry basis moisture content at time point i, %; m_1_ is the initial mass of the sample, g; m_2_ is the mass of the sample when it is dried to constant weight, g; m_i_ is the mass of the sample dried to time point i, g; m_0_ is the mass of the sample after complete drying, g.

### 2.3. Color

The color was determined following the methodology of Huang et al. [17], with slight modifications. The color was measured using a colorimeter (CS-580A, Eltra, Neuss, Germany), and the color was expressed by L*, a*, and b*. Before the measurement, the colorimeter was calibrated with a standard white plate. In addition, the color difference was expressed by ΔE, and the calculation Formula (3) was as follows:

(3) $$\Delta E\;=\sqrt{{\left(L_{i}^{*}-L_{0}^{*}\right)}^{2}\;+{\left(a_{i}^{*}-a_{0}^{*}\right)}^{2}\;+\;{\left(b_{i}^{*}\;-\;b_{0}^{*}\right)}^{2}}$$

Note: The subscript “0” is the color of the fresh WBS sample.

### 2.4. Texture

The method of Zheng et al. (2024) was used with some modifications [18]. The P/2E probe was used for the measurement, with a trigger force of 10 g, a deformation of 6 mm, and a downward speed of 2 mm/s. During the penetration test, the first peak was recorded as hardness.

### 2.5. Rehydration Ratio

The rehydration ratio was analyzed according to previously established methods [19]. A total of 3 g of the sample was accurately weighed and soaked in a water bath at 75 °C for 100 min. During the rehydration process, the sample was gently stirred with a glass rod to promote mass transfer. The rehydrated sample was taken out at intervals of 10 min, and the remaining water on the surface was removed with absorbent paper, weighed, and then put into water. The calculation formula (4) of the rehydration ratio is as follows:

(4) $$\mathrm{RR}(\%)\;=\frac{m_{4}}{m_{3}}\;\times \;100\%$$

Note: m_4_ is the mass of the sample after rehydration, g; m_3_ is the mass of the sample before rehydration, g.

### 2.6. Determination of Vitamin C Content

The vitamin C was determined following the methodology of Yue et al. (2025), with slight modifications [20]. An amount of 10 g of the sample was weighed and ground with 5 mL of 2% oxalic acid solution. WBS solids were filtered and removed. The filtrate was diluted with 2% oxalic acid to 100 mL. A total of 10 mL of 0.1 mg/mL standard vitamin C solution was taken into an evaporating dish and titrated with 0.2 mg/mL 2,6-dichlorophenol indophenol solution until a pink color was developed and did not fade within 30 s. The number of milligrams of vitamin C equivalent to 1 mL of dye was calculated. Then, 10 mL of the test solution was taken into an evaporating dish and titrated with the calibrated 2,6-dichlorophenol indophenol solution until a pink color was obtained that did not fade within 30 s. The amount of dye used was recorded. The vitamin C content was calculated according to formula (5).

(5) $$W\;\left(\mathrm{mg}/100\;g\right)=\frac{\left(y_{0}-y_{1}\right)\;\times \;A}{w_{0}}\;\times \;\frac{Z}{X}\;\times \;100$$

Note: W is the number of milligrams of vitamin C in 100 g of sample, mg/100 g; y_1_ is the number of milliliters of dye used for titration of blank, mL; y_0_ is the number of milliliters of dye used for titration of sample, mL; A is the number of milligrams of vitamin C equivalent to 1 mL of dye solution, mg/mL; w_0_ is the number of grams of sample, g; X is the volume of sample solution absorbed during titration, mL; Z is the total volume of sample solution after titration, mL.

### 2.7. Determination of Total Phenol Content

The total phenol content was analyzed according to previously established methods [21]. An amount of 0.2 g of sample was weighed and added to 25 mL of 80% methanol solution. The mixture was then placed in a water bath at 40 °C for 3 h, filtered, and diluted to 50 mL with 80% methanol solution. An amount of 500 μL of the extract was taken and mixed with 250 μL of 50% Folin phenol colorimetric agent. The resulting solution was mixed using a vortex for 5 min; then, 500 μL of 5% Na_2_CO_3_ solution was added. After being shaken well and protected from light for 60 min, the absorbance was measured at 765 nm. The results were expressed as milligrams of gallic acid equivalent (GAE) per gram of dry weight of the extract (mg GAE/g).

### 2.8. Microstructure Analysis

The method of Kamkari et al. (2024) was used to calculate the microstructure of dried WBSs with minor modifications [22]. Dried WBS stems were cut into 0.5 × 0.5 × 1 cm^3^ cuboids and fixed on a sample stage for metallization. High-resolution images were collected at 200 μm at 15 kV using a scanning electron microscope (TM4000Plus Tabletop Microscope, Hitachi High-Tech (Shanghai) International Trading Co., Ltd., Shanghai, China).

### 2.9. Browning Degree Analysis

The browning degree of the WBSs was analyzed using the previously established method, with minor modifications [23]. An amount of 0.2 g of WBS powder passing through a 60-mesh sieve was weighed and dissolved in 8 mL of 2% (*v*/*v*) acetic acid solution for 2 h. The solution was homogenized for 1 min, centrifuged at 10,000 r/min for 10 min, and the supernatant was collected. The absorbance at 420 nm was measured using a UV-Vis spectrophotometer (UV-1900i, Shimadzu Corporation, Kyoto, Japan). The 2% (*v*/*v*) acetic acid solution was used as a control group. Browning degree is expressed as absorbance units (AU) per gram of sample in AU/g.

### 2.10. Transverse Magnetic Relaxation Time (T2)

The method of Song, Yue, Gu, and Yang (2022) was used to test the transverse magnetic relaxation time of WBSs with minor changes [24]. The sample was placed in a sample tube, and the transverse relaxation time (T2) was measured using a CPMG (Car–Purcell–Meiboom–Gill) sequence. The sequence parameters were set as follows: main frequency 21 MHz; frequency offset 156,724.03 Hz; 90-degree pulse width 13.52 μs; sampling frequency 333.33 kHz; analog gain 20 dB; accumulation times 8; echo time 0.2 ms; and number of echoes 12,000.

### 2.11. Volatile Organic Compounds

The method of Meng et al. (2025) was used to test the intensity of volatile organic compounds in dried WBSs with minor changes [25]. A total of 3 g of dried WBSs was transferred to a headspace vial for HS-GC-IMS (FlavorSpec^®^, Dortmund, Germany). The experimental parameters of IMS were set as follows: the sample was incubated at 60 °C for 20 min, the column was FS-SE-54-CB-1 (15 m × 0.53 mm, 1 μm film thick), and the carrier gas (99.99% pure nitrogen) flow rate was 2 mL/min in the first 2 min, linearly increased to 10 mL/min within 2–10 min, and linearly increased to 100 mL/min within 10–20 min. Finally, IMS data were collected in positive mode and analyzed using VOCal software (v.1.0.7, G.A.S., Dortmund, Germany). The NIST 23 mass spectral library was used in combination with the retention index (RI), IMS database, and drift time (Dt) for qualitative analysis of volatile organic compounds. RI was calculated by the retention time of n-ketone C_4_–C_9_ (Sinopharm Chemical Reagent Beijing Co., Ltd., Beijing, China). The intensity of volatile organic compounds was based on the peak volume of the selected signal.

### 2.12. Statistical Analysis

In the experiment, there were five parallels in each group, and the results were expressed as mean ± standard deviation. Duncan’s variance analysis was performed on the experimental data using a one-way analysis of variance in SPSS 20 software, and the significance level was set at *p* < 0.05. Origin 2018 software was used to draw bar graphs and line graphs. The PCA and PLS-DA of volatile organic compounds were calculated using the metabolic analysis tool (https://www.metaboanalyst.ca/, 6 February 2025).

## 3. Results and Discussion

### 3.1. Drying Time Analysis

**Figure 1:**
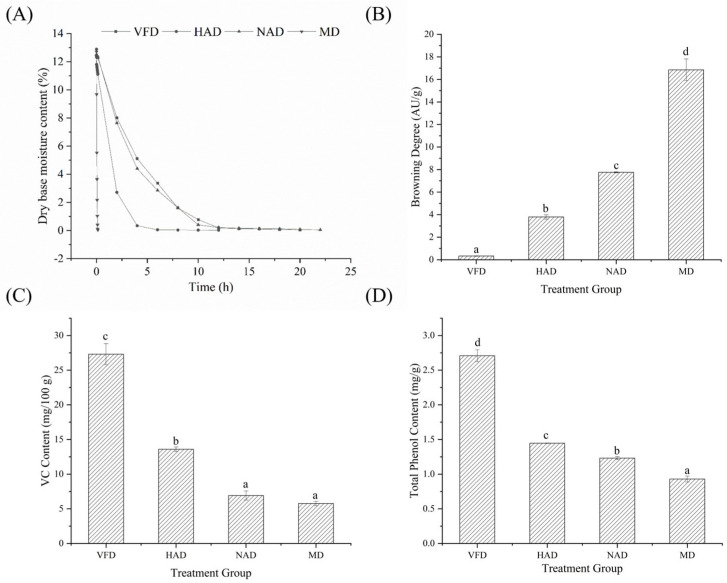
Analysis of the drying curve, browning degree, and nutritional value of dried WBS. (**A**) Drying curves of WBSs. (**B**) Browning degree of dried WBSs. (**C**) Vitamin C (VC) content of dried WBSs. (**D**) Total phenol content of dried WBSs. Different lowercase letters (a–d) indicate significant differences among groups (*p* < 0.05).

The dry basis moisture content curve is used to reflect the moisture changes during the drying process. The drying curves of WBSs under four different drying methods are shown in Figure 1A. The dry basis moisture content showed a downward trend with the extension of drying time, and the rate of moisture reduction exhibited a trend from a rapid decline at the beginning to a slower reduction later. Among all groups, the MD group required only 8 min for drying; the HAD group took 12 h; the VFD group took 20 h; and the NAD group took the longest drying time, lasting 22 h. The high frequency alternating electric field causes rapid friction and collision between water molecules, thereby accelerating the evaporation of water and shortening the drying time of the MD group [26]. Li et al. (2024) investigated the drying process of fermented Chinese cabbage and found that hot air drying (4 h) required significantly less time compared to vacuum freeze drying (18 h) [5]. A comparative study on different drying methods for *Solanum nigrum* leaves revealed that microwave drying achieved a faster drying rate than hot air drying, which was consistent with the results of this study [27]. The accelerated drying speed in microwave drying can be attributed to simultaneous internal and external heating of the material, promoting rapid water evaporation. Although microwave drying is efficient, it may lead to overheating when the temperature reaches a critical level. Excessive microwaves exposure can cause thermal accumulation within the material, reduce the evaporation rate, and potentially result in charring. Additionally, production costs should be taken into account. In our experiments, the overall drying expenses of the four drying methods can be ranked as VFD > HAD > MD > NAD based on the electricity costs observed. However, drying energy consumption can vary with both the drying method and sample specifications, necessitating future optimization of production processes to reduce costs [28].

### 3.2. Effect of Drying Methods on the Texture, Color, and Browning Degree of Dried WBSs

The fracture force, to a certain extent, reflected the quality attributes of the dried product. The textural properties of the dried WBSs are presented in Table 1. The fracture force of the VFD group was significantly lower than that of the other treatment groups (*p* < 0.05). This phenomenon can be attributed to the absence of volumetric compression during VFD, which results in reduced capacity to absorb and distribute external forces, thereby lowering resistance to fracture [29]. In contrast, the NAD group demonstrated significantly higher fracture force compared to all other groups (*p* < 0.05), potentially due to severe shrinkage of the material during drying, which increased fracture force [30]. The fracture force of the HAD group was significantly lower than that of the NAD group (*p* < 0.05), but still significantly higher than that of the MD group. This variation can be attributed to differences in volume shrinkage of the WBSs following application of the respective drying methods.

Color serves as a critical indicator of food quality and plays a significant role in influencing consumer purchasing behavior. Table 1 presents the color parameters of the dried WBSs. A higher color difference value indicated a greater deviation from the original fresh sample. Enzymatic browning and the Maillard reaction were identified as the primary factors influencing the drying quality of WBSs. Studies have shown that browning significantly reduced the L* value of food and exhibited a positive correlation with increases in both the a* and b* values [31]. Following the drying process, the brightness values of all WBS samples decreased, with the HAD and VFD groups exhibiting significantly higher brightness values than the other treatment groups. At the same time, in comparison to the CR and VFD groups, the a* and b* values in the NAD, HAD, and MD groups significantly increased, contributing to a higher color difference value. In conjunction with the browning analysis of dried WBSs (Figure 1B), the VFD group exhibited significantly lower browning than the other treatment groups (*p* < 0.05), at 0.33 AU/g, whereas the MD group exhibited the highest level of browning (*p* < 0.05), at 16.85 AU/g. The alteration in surface color of materials in the VFD group is mainly attributed to enzymatic browning, which results from the interaction between phenolic substrates and polyphenol oxidase on the cut surface of WBSs [32]. Heating and microwave energy increased the material temperature, thereby accelerating the browning process. An extended enzymatic reaction was primarily responsible for the increased color difference in the NAD group. Therefore, vacuum freeze drying showed better performance in preserving the texture and color of the dried WBSs while effectively inhibiting browning.

### 3.3. Effect of Drying Methods on the VC and Total Phenol Content in Dried WBSs

Vitamin C (VC) is highly sensitive to heat, oxygen, and light; therefore, the retention of VC in dried products serves as a critical indicator for evaluating the quality of dried fruits and vegetables. As illustrated in Figure 1C, the VC retention rate in the MD group was the lowest at 5.76 mg/100 g. This is likely attributed to the high internal temperature generated during microwaving drying, which promotes the degradation of VC [33]. The VC retention rate in the NAD group was 6.19 mg/100 g, potentially due to prolonged exposure to ultraviolet (UV) radiation, which can lead to the inactivation of VC [34]. In contrast, the VFD group showed the highest VC retention rate, significantly higher than the other treatment groups (*p* < 0.05), reaching 27.30 mg/100 g. This could be attributed to the low-temperature and vacuum conditions during freeze drying, which effectively minimize the loss of bioactive compounds and protect the oxidation-sensitive components. Therefore, the drying temperature is one of the main factors influencing VC content, with higher temperatures and longer exposure times resulting in greater VC degradation. Furthermore, a negative correlation was observed between vitamin C (VC) content and the degree of browning, which can be explained by the antioxidant properties of VC. The VFD group displayed the lowest browning degree (*p* < 0.05) and, accordingly, the highest VC content (*p* < 0.05). In contrast, the MD group showed the opposite trend, with the lowest VC content and the most severe browning (*p* < 0.05). A similar pattern was observed in the HAD and NAD groups, which exhibited intermediate trends consistent with the overall inverse relationship.

Phenolic compounds represent important natural active substances with strong antioxidant properties. However, similar to VC, they are also highly susceptible to oxidation and degradation under the influence of temperature, oxygen, and other environmental factors. The effect of different drying methods on the total phenol content in dried WBSs is presented in Figure 1D. The total phenol content in the vacuum freeze-dried group (2.71 mg/g) was significantly higher than that in the other treatment groups (*p* < 0.05). This phenomenon can be explained by the vacuum and low-temperature conditions, which suppress the activity of polyphenol oxidase and other oxidases, potentially leading to their inactivation [35]. The resulting reduction in enzymatic activity mitigates chemical interactions between phenolic compounds and vitamin C, thereby improving the retention of total phenols and vitamin C. In contrast, the reduced total phenol content observed in the MD (0.93 mg/g), HAD (1.45 mg/g), and NAD (1.23 mg/g) groups was likely associated with factors such as temperature, oxygen, and ultraviolet radiation [36,37,38]. Therefore, vacuum freeze drying was effective in improving the retention of both VC and total phenolic content. Similarly, total phenolic content was also negatively correlated with browning degree, consistent with the role of phenolic compounds as major substrates in browning reactions. The VFD group maintained the highest total phenolic content (*p* < 0.05) along with the least browning. Total phenolic levels decreased progressively across the HAD, NAD, and MD groups, while the browning degree increased in the same order, further supporting the inverse association between phenolic content and browning development.

### 3.4. Effect of Drying Method on Rehydration Ratio, Microstructure, and Water Distribution of Dried WBS

**Figure 2:**
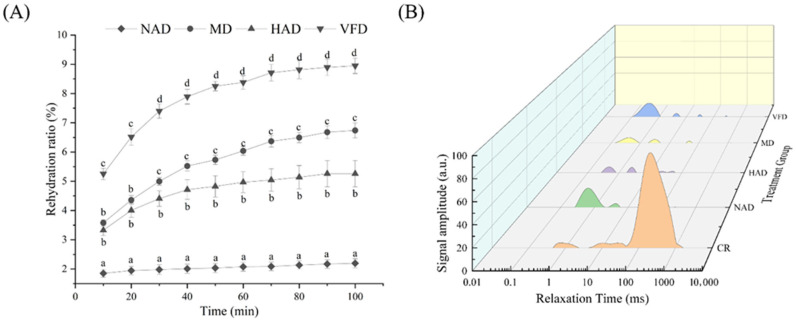
Rehydration ratio and T2 relaxation analysis of dried WBSs. (**A**) Rehydration ratio. (**B**) T2 relaxation. The different lowercases (a–d) indicate significant differences (*p* < 0.05).

**Figure 3:**
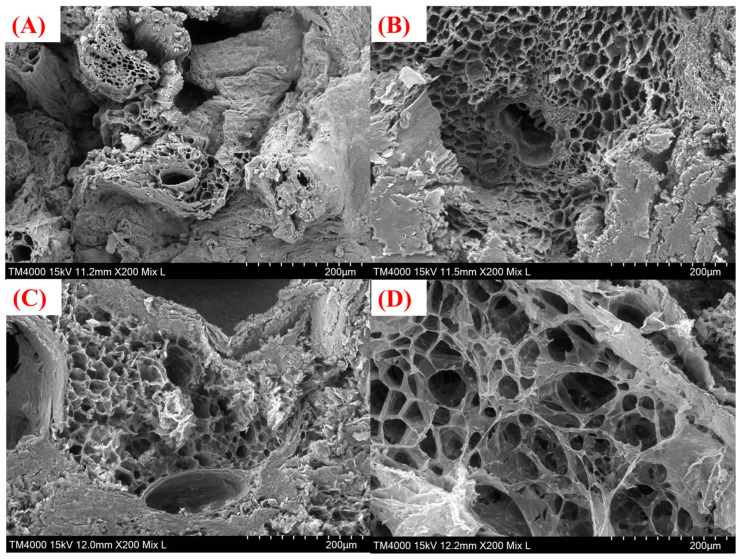
Microscopic structural analysis of dried WBSs (200× magnification). (**A**) NAD group. (**B**) HAD group. (**C**) MD group. (**D**) VFD group.

The rehydration performance of dried products serves as a crucial quality indicator for evaluating dried fruits and vegetables [39]. As shown in Figure 2A, the VFD group exhibited the highest rehydration ratio, which was significantly higher than that of other treatment groups (*p* < 0.05), followed by the MD group and the HAD group, while the NAD group had the lowest rehydration ratio. This variation is closely associated with differences in microstructure characteristics. At the 20th minute, the rehydration ratio of each group dropped rapidly and then tended to be flat. Microstructural analysis of the dried WBSs (Figure 3) revealed that the samples in the NAD group (Figure 3A) underwent severe shrinkage and extensive structural damage, which likely contributed to their reduced rehydration ratio [40]; the WBS samples in the HAD group (Figure 3B) exhibited an obvious shrinkage, and the gaps between the cell walls shrank due to uneven water loss during the hot air drying and dehydration process [3]. The MD group (Figure 3C) also showed a slight shrinkage, potentially resulting from non-uniform microwave heating [41]. In contrast, the VFD group (Figure 3D) was dried by water sublimation to retain a rich porous structure [42]. Therefore, vacuum freeze drying has the best microstructural characteristics, which contribute to its superior rehydration performance.

Low-field nuclear magnetic resonance (NMR) is a widely utilized technique for analyzing the distribution and relative proportion of various bound water states within a sample. T2 relaxation, defined as the transverse component of magnetization, constitutes a fundamental parameter in this type of analysis. The transverse relaxation time, which is the duration of the transverse magnetization relaxation, may differ depending on the specific water types present in plant cells [43]. As demonstrated in Figure 2B, three distinct peaks were observed and subsequently classified into three distinct water categories. Specifically, T2b (0–10 ms) corresponds to water tightly bound to macromolecular particles (i.e., bound water), T21 (10–100 ms) represents water retained within the cytoplasm, and T22 (100–1000 ms) reflects water with high mobility located in vacuoles and intercellular spaces (i.e., free water) [44]. A comparison analysis of the four drying methods with fresh samples revealed that all methods significantly reduced the amount of free water, thereby confirming the effectiveness of the drying treatments. Notably, the bound water content increased in the NAD and VFD groups. During the drying process, hydrophilic substances such as proteins and polysaccharides become exposed, exhibiting pronounced hygroscopic properties and the capacity to bind with water molecules. This binding process leads to an increase in the amount of water that can be adsorbed, resulting in an elevated bound water content [45]. Therefore, the bound water in the NAD group may originate from the conversion of free water, whereas in the VFD group, it may be attributed to moisture absorption from the surrounding air.

### 3.5. Impact of Drying Methods on the Volatile Organic Compounds in Dried WBSs

**Figure 4:**
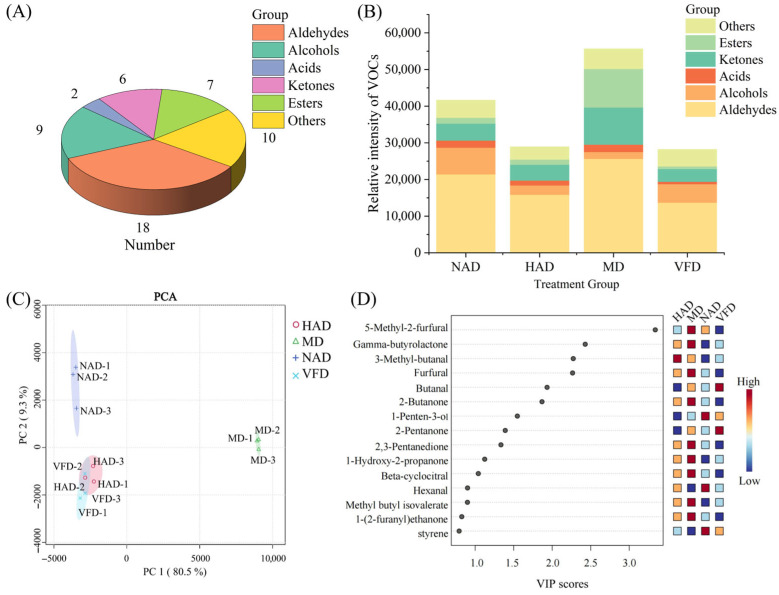
Analysis of volatile organic compounds in dried WBSs. (**A**) Classification of VOCs. (**B**) Total relative intensity of VOCs in dried WBSs. (**C**) PCA of VOCs in dried WBSs. (**D**) PLS-DA analysis of VOCs in dried WBSs.

The volatile organic compounds (VOCs) in dried WBSs during the drying process were determined using the GC-IMS method. Detailed VOC results are provided in Table S1. A total of 52 VOCs were identified in dried WBS, including 18 aldehydes, 9 alcohols, 2 acids, 6 ketones, 7 esters, and 10 other compounds (Figure 4A).

Aldehydes were primarily derived from the degradation of amino acids and the Maillard reaction, and due to their low flavor thresholds, they became significant contributors to the overall aroma profile of dried WBSs [46]. The variation trends of the aldehydes were unstable among the groups. 3-methyl-butanal was the aldehyde with the highest relative intensity in the NAD (3583.69 ± 73.05), HAD (5176.42 ± 29.99), and VFD (3743.09 ± 132.76) groups. Additionally, the relative intensity of 3-methyl-butanal in the MD group was also substantial at 4717.30 ± 38.90, with significant differences were observed among all groups (*p* < 0.05). 3-methyl-butanal may be an inherent VOC in WBSs, contributing to an apple-like aroma and positively affecting the overall flavor of WBSs. 5-Methyl-2-furfural exhibited the highest relative intensity in the MD group (8065.77 ± 59.36), which was significantly higher compared to other drying treatment groups (*p* < 0.05). This compound imparts a caramel-like aroma to dried WBSs, potentially associated with the increased browning degree in the MD group.

Alcohol compounds are primarily derived from the oxidation of lipids and the degradation of amino acids, which contribute to the development of desirable aromatic profiles in dried WBSs [47]. In the NAD group, 1-Penten-3-ol exhibited the highest relative intensity at 1790.12 ± 60.17, significantly higher than in other treatment groups (*p* < 0.05), and imparted a distinct fruity aroma to the final product. In the HAD (771.40 ± 2.97) and VFD (1401.26 ± 46.68) groups, 3-methylbutanol was the alcohol compound with the highest relative intensity, endowing dried WBSs with a malty aroma [48]. In addition, the 3-methylbutanol in the NAD group was slightly different from that in the HAD group, which may be related to the volatilization behavior of alcohol substances during the drying process. In the MD group, ethanol exhibited the highest relative intensity at 627.67 ± 26.02; however, ethanol also displayed relatively high intensity in the NAD group (1497.30 ± 71.76), which was significantly higher compared to other treatment groups (*p* < 0.05).

Acid compounds were primarily derived from the degradation of sugars [49]. Only two acid VOCs were identified, namely acetic acid and propionic acid. In all treatment groups, the relative intensity of acetic acid was higher than that of propionic acid; however, its pungent aroma might have been detrimental to the overall aroma profile of dried WBSs. Notably, the acetic acid content in the MD group was significantly higher than that in the other groups (*p* < 0.05), at 1486.18 ± 55.07. This phenomenon could be attributed to the Maillard reaction between sugars and proteins or amino acids during the drying process, which is known to generate acetic acid. This is consistent with the highest degree of browning observed in the MD group [50].

Ketone compounds contributed unique aromatic characteristics to dried WBSs and are primarily derived from lipid oxidation and amino acid degradation [51]. In the NAD group (1361.14 ± 31.7) and the HAD group (1419.84 ± 53.48), 2-butanone exhibited the highest relative intensity (*p* > 0.05). In addition, the 2-butanone in the MD group also had a higher relative intensity of 1963.42 ± 54.88, which was significantly higher than that in the other treatment groups (*p* < 0.05). The relative intensity of 2-butanone in the VFD group was the lowest (*p* < 0.05), which may be related to its volatility. Additionally, 2-pentanone demonstrated a relatively high intensity in the NAD group at 1354.49 ± 63.27, and was identified as the predominant ketone compound in the VFD group. This may be due to the higher drying temperature promoting the volatilization of 2-pentanone, which decreases its relative intensity in the HAD and MD groups. Both 2-butanone and 2-pentanone possessed an aroma similar to acetone and were characterized as fruity, thereby contributing positively to the overall aroma profile of dried WBSs. In the MD group, 1-Hydroxy-2-propanone demonstrated the highest relative intensity at 2448.20 ± 84.37, which was significantly higher than that observed in other treatments groups (*p* < 0.05). The relative intensity of 1-Hydroxy-2-propanone in the MD group (2448.2 ± 84.37) was significantly higher than that in the other treatment groups (*p* < 0.05), which may be related to the higher browning degree in the MD group. Similarly, 2,3-Pentanedione in the MD group also showed a high relative intensity at 2354.83 ± 28.82, and together with 1-Hydroxy-2-propanone, it provided sweetness and a caramel-like aroma to dried WBSs.

Acid–alcohol esterification represents the primary reaction pathway for the formation of ester compounds, and the relatively high temperature during the drying process also facilitated this reaction [52]. As shown in Figure 4B, compared with the VFD group, the NAD, HAD, and MD groups exhibited a higher relative intensity of esters, which was attributed to the temperature variations during the drying process. In the NAD group, Butyl formate exhibited the highest relative intensity at 770.74 ± 17.15, imparting a fruity aroma to the dried WBS. Methyl acetate was identified as the ester compound with the highest relative intensity in the HAD group (385.25 ± 0.83) and was characterized by a fruity aroma. In addition, the relative intensities of Butyl formate and Methyl acetate in the VFD group were significantly lower than those observed in the other treatment groups (*p* < 0.05), which may be related to inhibiting the esterification reaction by low temperature. Gamma-butyrolactone was identified as the ester with the highest relative intensity in both the MD and VDF groups. The relative intensity of the MD group (5891.98 ± 65.27) was significantly higher than that of the other treatment groups (*p* < 0.05). Gamma-butyrolactone was described as possessing a creamy aroma, which appeared to benefit the overall aroma of dried WBSs.

An additional 10 types of VOCs were identified. In the NAD group, styrene exhibited the highest relative intensity at 1212.38 ± 43.03, imparting an aromatic fragrance to dried WBSs. Meanwhile, Anisole was identified as the VOC with the highest relative intensity in the HAD (1325.53 ± 36.46), MD (1229.11 ± 13.21), and VFD (1456.15 ± 70.92) groups. It was characterized by a sweet, anise-like aroma.

To obtain a more comprehensive understanding of the effects of different drying techniques on the VOC composition of dried water bamboo, principal component analysis (PCA) (Figure 4C) and partial least squares discriminant analysis (PLS-DA) (Figure 4D) were performed based on the raw data. As shown in Figure 4C, the variations explained by PC1 and PC2 were 80.5% and 9.3%, respectively. Along with PC1, the MD group was located in the positive quadrant, while the NAD, HAD, and VFD groups were in the negative quadrant. With respect to PC2, the NAD and MD groups were found in the positive quadrant, whereas the HAD and VFD groups were observed in the negative quadrant. Moreover, the VOC profiles of the HAD and VFD groups were the most similar ones in terms of relative intensity. A total of eleven volatile organic compounds exhibited significant differences among the treatment groups (VIP ≥ 1, *p* < 0.05), including five aldehydes (furfural, β-cyclocitral, 5-methyl-2-furfural, 3-methyl-butanal, and butanal), four ketones (1-hydroxy-2-propanone, 2,3-pentanedione, 2-butanone, and 2-pentanone), one alcohol (1-penten-3-ol), and one ester compound (γ-butyrolactone). The PCA combined with PLS-DA indicated that PC1 likely captured a significant portion of the variation in the relative intensities of certain VOCs. Samples with higher relative intensities of 5-methyl-2-furfural, γ-butyrolactone, furfural, 2-butanone, 2,3-pentanedione, 1-hydroxy-2-propanone, and β-cyclocitral had positive scores along PC1, whereas those with lower relative intensities had negative scores. This was the reason the MD group was located in the positive quadrant of PC1. Similarly, PC2 also captured substantial variability among VOCs; samples with higher relative intensity of 3-methyl-butanal showed positive scores, while those with lower intensity of 1-penten-3-ol also showed positive scores—conversely, lower intensity of 3-methyl-butanal and higher intensity of 1-penten-3-ol were associated with negative scores. This was also the reason HAD and MD were located in the positive quadrant of PC2. Ultimately, this resulted in the MD group being positioned in the first quadrant, the NAD group in the second quadrant, and VFD and HAD in the third quadrant, demonstrating the impact of different drying methods on the variation in VOC composition.

### 3.6. The Fingerprint Map Analysis

**Figure 5:**
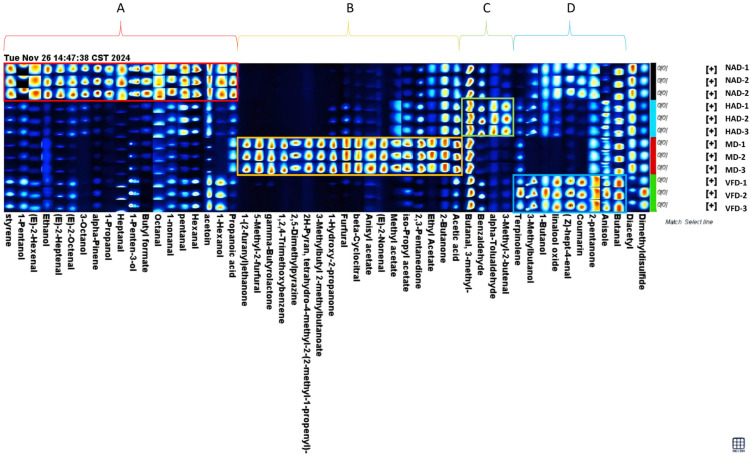
Fingerprint map analysis of VOCs in dried WBS. The zone of each topographic plot containing most of the critical data is labeled with a red rectangle.

Figure 5 provides an overview of dried WBSs after different drying methods. The higher the aroma intensity of a volatile organic compound (VOC), the brighter the corresponding block appeared in the figure. Four distinct regions, A through D, were identified in Figure 5. Region A comprised 19 VOCs, primarily aldehydes and alcohols, whose relative intensities were significantly higher in the NAD group compared to the other treatment groups (*p* < 0.05). Among them, pentanal exhibited the highest relative intensity at 2915.85 ± 63.91 and was therefore considered a potential marker compound for the NAD group, bringing a pungent odor. Region B mainly represented 18 VOCs in the MD group that were significantly elevated compared to the other groups (*p* < 0.05), consisting predominantly of aldehydes, ketones, and esters. The relative intensity of 5-methyl-2-furfural was the highest in this group, reaching 8065.77 ± 59.36, suggesting it as a potential marker compound for the MD group. High levels of 5-methyl-2-furfural in the MD group lead to excessive browning flavor. Region C included VOCs that were significantly higher in the HAD group, comprising four aldehydes: 3-methyl-2-butenal, 3-methyl-butanal, benzaldehyde, and α-tolualdehyde, which may have originated from Maillard or browning reactions. Among these, 3-methyl-butanal showed the highest relative intensity at 5176.42 ± 29.99 and could be a potential marker for the HAD group. Region D consisted of nine VOCs, primarily associated with the VFD group, among which butanal exhibited the highest relative intensity at 3875.01 ± 153.92. The high relative intensity of butanal was found in the VFD group, which indicates its potential as a marker compound for the pungent aldehyde odor associated with the VFD group.

## 4. Conclusions

Food dehydration has increasingly become a crucial process for extending the shelf life of WBSs. This study investigated the effects of four drying techniques—natural air drying (NAD), hot air drying (HAD), microwave drying (MD), and vacuum freeze drying (VFD)—on the drying quality and volatile organic compounds (VOCs) of WBS. T2 relaxation results demonstrated that all four drying techniques effectively removed free water, thereby achieving efficient dehydration. Compared to the other drying methods, MD significantly shortened the drying time. However, it also resulted in a higher browning degree (16.85 AU/g), lower retention of vitamin C (5.76 mg/100 g), and lower total phenolic content (0.93 mg/g). In contrast, VFD, leveraging its vacuum and low-temperature environment, produced dried WBSs with a browning degree (0.33 AU/g) and color difference (8.98) significantly lower than the other techniques. Concurrently, the VFD group exhibited significantly higher retention rates of vitamin C (27.30 mg/100 g) and total phenolics (2.71 mg/g) compared to the other drying treatment groups. This endowed VFD-treated WBSs with superior appearance and nutritional value. Both MD and VFD treatments resulted in dried WBSs with abundant porous structures, which not only enhanced the textural properties but also improved rehydration capacity. Gas chromatography–ion mobility spectrometry (GC-IMS) identified a total of 52 VOCs. Among these, 3-methyl-butanal exhibited the highest relative intensity in the NAD, HAD, and VFD groups, whereas 5-methyl-2-furfural was the VOC with the highest relative intensity in the MD group. The VOC profiles of dried WBSs varied considerably depending on the drying technique applied, with 5-methyl-2-furfural (VIP > 3.0) identified as the primary contributor to these differences. Characteristic VOCs varied among the drying techniques: 3-methyl-butanal (5176.42 ± 29.99) was proposed as a marker compound for the HAD group; butanal (3875.01 ± 153.92) for the VFD group; pentanal (2915.85 ± 63.91) for the NAD group; and 5-Methyl-2-furfural (8065.77 ± 59.36) for the MD group. In conclusion, VFD demonstrated superior performance in preserving both the drying quality and nutritional attributes of dried WBSs, with butanal established as its characteristic VOC. This research provided a theoretical foundation for the industrial implementation of VFD to maintain quality stability during WBS drying.

## Figures and Tables

**Table 1:** Effects of different drying methods on the texture and color of dried WBS.

Drying Method	Fracture Force (g)	L*	a*	b*	ΔE
CR	438.00 ± 17.08 b	85.52 ± 1.24 c	−0.82 ± 0.04 a	15.74 ± 0.21 a	/
HAD	1667.78 ± 3.71 d	74.64 ± 2.93 b	3.45 ± 0.30 b	21.55 ± 0.75 c	13.05
MD	1009.18 ± 52.71 c	41.66 ± 2.51 a	8.76 ± 0.42 c	17.76 ± 0.37 b	44.94
VFD	196.71 ± 4.43 a	76.57 ± 2.74 b	−0.77 ± 0.13 a	15.04 ± 0.65 a	8.98
NAD	1815.62 ± 0.50 e	41.88 ± 0.93 a	9.47 ± 0.48 d	23.00 ± 0.91 d	45.41

Note: different lowercase letters (a–e) indicate that there are significant differences among the treatment groups (*p* < 0.05).

## Data Availability

The original contributions presented in this study are included in the article. Further inquiries can be directed to the corresponding authors.

## Supplementary Materials

The following supporting information can be downloaded at https://www.mdpi.com/article/10.3390/foods14244357/s1, Table S1: The effect of different drying methods on VOCs in dried WBSs.

## Abbreviations

The following abbreviations are used in this manuscript:

WBS	Water bamboo shoots
NAD	Natural air drying
HAD	Hot air drying
MD	Microwave drying
VFD	Vacuum freeze drying
VOC	Volatile organic compound
GC-IMS	Gas chromatography–ion mobility spectrometry
